# Bridging the Quality-Price Gap: Unlocking Consumer Premiums for High-Quality Rice in China

**DOI:** 10.3390/foods14071184

**Published:** 2025-03-28

**Authors:** Yiyuan Miao, Junmao Sun, Rui Liu, Jiazhang Huang, Jiping Sheng

**Affiliations:** 1 School of Agricultural Economics and Rural Development, Renmin University of China, Beijing 100872, China; miaoyiyuan@ruc.edu.cn; 2 Institute of Food and Nutrition Development, Ministry of Agriculture and Rural Affairs, Beijing 100081, China; sunjunmao@caas.cn (J.S.); liurui@caas.cn (R.L.)

**Keywords:** premium pricing, consumer preferences, rice quality, willingness to pay, multi-stage decision, hedonic pricing

## Abstract

The transition of global agriculture from yield-driven production to quality-driven systems has gained urgency, where premium pricing strategies offer pathways to enhance farmer incomes and promote sustainable practices. As a critical staple crop, rice exemplifies the challenges of aligning producer standards with consumer preferences to realize market premiums. This study systematically evaluates determinants of consumers’ willingness to pay (WTP) for premium rice, integrating analyses of attribute preferences, cognition perception, and purchasing experience. Utilizing survey data from 1714 consumers across four Chinese cities, we employ principal component analysis to identify key quality dimensions and ordered logit models to quantify their impacts. Hedonic pricing theory informs the estimation of implicit prices for specific attributes. The results reveal that intrinsic characteristics (like nutrition) and extrinsic cues (like the brand), along with consumers’ nutritional awareness, knowledge, and perceptions of quality-price correlation, jointly drive premium WTP. The mean acceptable premium reaches 4.52 yuan/500 g, with nutritional attention enhancements commanding the highest valuation (0.171 yuan/500 g). The findings underscore the necessity of standardized quality grading systems aligned with consumer preferences and targeted interventions to bridge information asymmetries. Policymakers are recommended to improve supply-side quality signaling through enhanced packaging and certification systems while strengthening demand-side nutrition education to facilitate value chain coordination and sustainable, high-quality development in agriculture.

## 1. Introduction

The principle of “premium pricing for premium quality” is integral to advancing the high-quality development of agriculture globally [1,2]. As the global economy expands, agricultural production has generally increased, providing a strong foundation for ensuring global food security [3]. From a worldwide agricultural development perspective, rising income levels and evolving consumption patterns have significantly heightened consumer demand for high-quality agricultural products [4]. Consequently, there is an urgent need for agricultural development to shift its focus from merely increasing yields to improving product quality [4,5]. This transition is not only a necessary response to changing market demands but also a critical path toward enhancing the overall competitiveness and sustainability of agriculture.

Premium pricing serves as a vital internal driver and safeguard for agricultural transformation and modernization [6]. The core of premium pricing lies in leveraging market mechanisms and pricing strategies to guide farmers in producing high-quality agricultural products, thereby fostering sustainable agricultural development. These high-quality products satisfy consumers’ demands for a superior lifestyle, consequently improving their quality of life. At the same time, the premium pricing mechanism allows farmers to achieve higher income returns, incentivizing further investment in the production of high-quality agricultural goods [7,8]. This dynamic not only enhances farmers’ incomes but also aligns with the growing consumer demand for high-quality agricultural products. For developing countries, premium pricing is a key strategy for modernizing agriculture and improving farmer incomes [9,10]. By securing higher prices for quality grains, farmers are motivated to enhance production standards, thus advancing the development of the grain industry.

Existing research on premium pricing in the global grain sector predominantly focuses on macro-level qualitative analyses, emphasizing theoretical frameworks and implementation challenges. A consensus has emerged that the primary barrier to premium pricing in agricultural markets is the imbalance between supply and demand [11]. Key factors influencing the alignment of premium agricultural product supply and demand include information asymmetry [12] and discrepancies between the quality attributes offered by producers and consumer preferences [13,14]. The quality improvements made on the production side do not always align with consumers’ willingness to pay a premium. Willingness to pay (WTP) is defined as the maximum price a customer is willing to pay for a product or service, reflecting their subjective valuation and personal assessment of its utility [15]. Therefore, gaining a deeper understanding of consumer preferences through calculating WTP, particularly regarding rice, is essential for enabling producers to recognize and meet the attributes that drive premium willingness. This is crucial for effectively aligning supply with demand and facilitating the successful implementation of premium pricing.

As one of the most vital agricultural commodities globally, the quality and pricing of rice directly influence global food security and living standards [16]. Rice, a staple food crop consumed worldwide, plays an essential role in promoting high-quality agricultural development [17]. This study aims to identify the key factors that promote the realization of premium pricing for rice. The research objectives are as follows: (1) to conduct a comprehensive investigation of consumer demands and preferences for rice attributes based on existing literature and standards, enhancing the consumer-based evaluation system for high-quality rice; (2) to analyze the factors influencing consumers’ willingness to pay a premium for high-quality rice, including rice quality attributes and demographic factors, thereby identifying constraints from the consumer side in achieving premium pricing; (3) to estimate acceptable premium payment levels among consumers and explore the relationships between various rice quality characteristics, consumer perceptions, and premium payment willingness, ultimately proposing demand-side pricing strategies.

The findings of this study can provide valuable insights for the formulation of differentiated subsidy policies in developing countries, such as production incentives for consumer-preferred quality attributes. These policies would support efforts to secure farmer incomes and guide agricultural systems toward higher quality and more sustainable development, thereby enhancing the overall effectiveness of agricultural advancement.

## 2. Conceptual Framework and Research Hypotheses

To address the research questions outlined, this study reviews existing literature and proposes a research framework from several perspectives.

### 2.1. Research Hypothesis

A significant barrier to achieving premium pricing is the misalignment between producers’ and consumers’ definitions of “quality” [18]. Various countries have established quality standards for rice, such as China’s GB/T 1354-2018 [19]. These standards provide benchmarks for quality assessment at the production level, focusing predominantly on the product’s physical attributes during processing [20,21]. However, they do not encompass critical consumer considerations such as taste and nutritional value, which play a crucial role in purchasing decisions [22,23]. Consequently, the existing quality ratings fail to facilitate price discrimination among consumers, indicating that the premiums established by producers are not reflected in market prices. This discrepancy may arise because consumers are often either unaware of these ratings or indifferent to the established standards [24,25]. Furthermore, these standards may not adequately reflect consumer preferences [18,26]. Therefore, an analysis of the factors affecting premium pricing should commence with an understanding of consumer-defined quality standards, specifically regarding their preferred attributes in rice products.

Drawing on hedonic pricing theory, a consumer’s willingness to pay for a product is influenced not only by the product itself but also by its specific attributes [27,28]. Hedonic Pricing Theory is an economic theory that determines the price of a good or service based on the subjective valuations consumers place on its various attributes or characteristics. This theory posits that the price of a product can be decomposed into the sum of the values of its individual attributes. Each attribute has an implicit price that reflects consumers’ preferences and willingness to pay for that specific feature [29]. Thus, consumer demand for specific product characteristics is fundamental to implementing a successful premium pricing strategy. By linking consumer preferences and willingness to pay with product attributes, a viable pricing model can be developed [30]. Employing characteristic pricing theory and related methodologies, an empirical study can elucidate the relationship between consumer preference for rice product attributes and their willingness to pay a premium. At the demand formation stage, the literature suggests that demand formation is influenced by cognitive levels, trust, and consumer preferences [31,32]. Consumers evaluate options based on the importance and assessment of product attributes [33,34]. Specifically, they may prioritize intrinsic features such as appearance, taste, and nutrition, in addition to extrinsic cues, including origin, brand, certification, and quality grade [35,36,37]. These external attributes often reflect intrinsic qualities, influenced by past purchasing experiences [32,38,39]. Thus, this study proposes the following hypotheses:

**H1:**  *Consumer preference for rice product attributes will significantly and positively influence their willingness to pay a premium.*

**H1a:**  *The importance assigned to intrinsic attributes (appearance, taste) will significantly and positively affect willingness to pay a premium.*

**H1b:**  *The importance assigned to extrinsic attributes (brand, origin) will significantly and positively affect willingness to pay a premium.*

From the perspective of supply-demand alignment, it is essential to understand the factors influencing consumer purchasing decisions, in addition to product attributes, to enable targeted interventions in consumer behavior [40,41]. Consumer decision-making theories suggest that this process involves multiple stages, including awareness formation, information search, option evaluation, purchase decision, and post-purchase evaluation [42,43,44].

During the awareness and evaluation stages, the Theory of Planned Behavior posits that consumer cognition and attitudes significantly influence decision-making [45]. Parashar et al. [46] demonstrated that positive consumer attitudes enhance purchase intentions, observing a strong correlation between organic food consumption and both health consciousness and environmental awareness. Conversely, Wang et al. [47] found that self-claims regarding geographic indication (GI) did not effectively generate price premiums, whereas GI certification retained its competitive advantage. Additionally, consumers’ perceptions of quality and their ability to recognize premium products may impact their purchase intentions [48,49]. Therefore, it is imperative to incorporate consumer interest and knowledge regarding related information as significant factors influencing the willingness to pay a premium. Therefore, this study posits the following hypotheses based on the Theory of Planned Behavior:

**H2:**  *Consumer cognitive perceptions will significantly and positively influence their willingness to pay a premium.*

Based on the Howard-Sheth model, the consumer decision-making process involves the stages of attention, understanding, attitude formation, purchase intention formation, and actual purchase behavior, and consumers will evaluate the alternatives according to their own needs and beliefs [50,51]. In the purchasing process, research indicates that previous purchasing experiences—particularly the accessibility of traceability information—and the perception that quality and price are interconnected [35] can significantly influence ongoing consumer behavior [52]. The perception of a quality-price relationship refers to consumers’ beliefs about the correlation between product quality and price, shaped by feedback from past purchasing experiences [53]. For instance, if a consumer, guided by the belief that “you get what you pay for”, purchases a high-priced item that turns out to be of poor quality, or if a lower-priced item outperforms a comparable, higher-priced one, their perception of the quality-price relationship diminishes [54]. Consequently, consumers become less confident in the correspondence between quality and price, which impedes the formation of a virtuous cycle in the market for high-quality, premium-priced goods. The trustworthiness of labels and overall consumer trust and satisfaction may vary in this way [55,56,57], emphasizing that consumer purchasing experiences significantly impact decision formation. Therefore, this study posits the following hypotheses based on the Howard-Sheth model:

**H3:**  *Consumer purchasing experiences will significantly and positively influence their willingness to pay a premium.*

### 2.2. Research Design

This study employs a “problem identification–mechanism analysis–strategy proposal” approach, drawing on previous research and identified influencing factors. A multi-stage, hierarchical model is utilized to identify factors that influence consumers’ willingness to pay a premium and to estimate reasonable premium ranges for specific attributes [3,57]. The empirical analysis is conducted in two phases, addressing both the determinants of willingness to pay and the components of premium pricing. A two-stage analytical framework was used, with the first stage identifying the key determinants of consumers’ willingness to buy higher-quality and higher-priced rice based on the three research hypotheses, and then the second stage being the estimation of the level of premiums for each specific attribute, i.e., how much consumers are willing to pay for that attribute.

The three hypotheses mentioned above were tested in the first stage, setting the stage for what influences should be considered in the second stage. In the second phase, further identification of the prices consumers are willing to pay for specific attributes is undertaken. Drawing from the literature and the retail realities of the rice industry, secondary indicators are developed. For the first hypothesis, an evaluation system is constructed that considers rice attributes, including appearance, taste, nutrition, breed, origin, brand, certification, and quality level. For the second factor, consumers’ attention to nutrient facts and quality grades, as well as their understanding of certification labels, is measured. Regarding the third factor, perceived quality-price relationships, trust in quality safety, and ease of information access are assessed, determining whether a price premium corresponds to quality improvement, trust in quality inspections, and the capacity to distinguish quality information.

The decision to analyze factors influencing both willingness to purchase (WTP) and the specific price consumers are willing to pay using a two-stage approach is driven by several key considerations.

Primarily, this design allows for enriching both the theoretical and empirical granularity of the analysis. The first stage serves to validate whether the core framework (H1–H3) significantly influences the decision to purchase premium rice, aligning with theoretical predictions. Subsequently, the second stage quantifies the incremental premium associated with actionable attributes, providing nuanced insights for policymakers and marketers by revealing, for example, how improving a unit of nutrition enhances WTP. This staged approach mirrors hedonic pricing studies that differentiate between the “whether” and “how much” questions, acknowledging that the factors influencing initial purchase intention may differ from those determining the final willingness to pay a specific price.

Furthermore, employing a two-stage approach helps mitigate the risks of multicollinearity and overfitting. The multitude of factors influencing WTP during the purchase decision process, which may exhibit hierarchical relationships, poses a challenge. A single-step model incorporating all 17 secondary indicators would likely encounter substantial multicollinearity, obscuring the independent effects of individual variables. To circumvent this issue, we first employ principal component analysis (PCA) to reduce dimensionality, isolating orthogonal factors, such as product preference and cognitive perceptions. These factors capture underlying constructs without overlapping variance, thereby ensuring robust parameter estimation in the ordered Logit model. This strategy aligns with established practices in consumer behavior research for managing the complexities associated with high-dimensional data.

Finally, a two-stage design enhances interpretability and policy relevance. A single, comprehensive model would conflate the identification of influential structural frameworks with the valuation of specific attributes, complicating the translation of results into actionable strategies. For example, while the first stage might confirm the importance of nutrition awareness (H2), the second stage reveals which specific enhancements to labeling yield the highest marginal returns. This dual focus informs both broad policy strategies and practical interventions. By first elucidating the importance of a factor and then targeting specific countermeasures, a more refined distinction can be made for certain scenarios. For instance, a consumer’s overall nutrition awareness may influence their willingness to purchase, but enhancing label literacy may not necessarily increase their maximum WTP, or the premium increment may be too small to warrant a recommendation. Therefore, this more rigorous two-stage design, balancing parsimony and precision, addresses the need for enriching theoretical granularity, managing data complexity, and enhancing practical applicability, representing a key contribution of this study compared to prior research.

## 3. Materials and Methods

### 3.1. Data Collection

This study targeted urban consumer groups across four cities in distinct geographical regions of China. The decision to focus on urban consumers was based on several considerations. First, urban populations typically possess higher incomes and a greater willingness to purchase premium rice, thereby representing the primary consumer demographic for such products and demonstrating a higher likelihood of survey participation. Second, rural consumers often engage in self-sustaining practices, consuming rice sourced from their production or of acquaintances, complicating assessments of their willingness to pay for rice. Third, urban areas generally host individuals with higher educational attainment, leading to increased receptivity to new concepts and heightened awareness of the quality attributes associated with premium rice. Therefore, data collected from urban regions are deemed more reliable and valid.

The four cities-Beijing, Luoyang, Wuhan, and Shenzhen-were selected based on a multi-criteria framework. Firstly, meet geographical representation. It is noted that distinct dietary habits exist between southern and northern China, exemplified by the adage “southern rice and northern wheat”. In this context, southern regions favor rice and rice products, whereas northern regions may prefer wheat-based foods, including noodles and bread [58]. Consequently, the inclusion of cities covering northern (Beijing), central (Luoyang), central-southern (Wuhan), and southern (Shenzhen) regions to capture dietary diversity. Secondly, to cover economic gradient. Significant disparities in income and consumption levels across cities impact the willingness and ability to pay for premium rice. Thus, cities at various stages of economic development were selected, with Beijing and Shenzhen classified as first-tier cities and Wuhan and Luoyang classified as second-and third-tier cities, covering different income groups. Thirdly, rice consumption culture and policy relevance. Beijing and Shenzhen were chosen due to substantial rice consumption with their large population. Additionally, Luoyang (in Henan Province) and Wuhan (in Hubei Province) were included as representative bases for wheat and rice cultivation, respectively, and as cities with significant rice consumption within their respective regions. Furthermore, all selected cities incorporate rice into their essential agricultural product security plans. Therefore, the current city selection encompasses diverse characteristics that potentially influence rice consumption intentions as shown in Table 1, ensuring comprehensive sample diversity. This selection strategy also enables comparisons of the potential impact of other characteristics while controlling for one factor; for example, the comparison between Beijing and Shenzhen allows for examining geographical influences while controlling income level.

A preliminary survey conducted in March 2021 served to refine the formal survey design, incorporating feedback regarding question formulation, logical flow, and language clarity. The final questionnaire consisted of four sections. The first section addressed respondents’ rice purchasing behaviors through four basic questions, such as purchasing frequency. The second section focused on evaluating consumer emphasis on eight key product attributes to validate H1, including appearance, taste, nutrition and others to measure the variable product preference; subsequently, consumers’ willingness to buy and premium price they would pay were measured, respectively. The third section aimed to validate H2 and H3 by employing six Likert-scale items assessing consumer perceptions and purchase experiences. The final section collected demographic characteristics of respondents, such as gender, age, household size and composition, as detailed in Table 2.

The formal survey was subsequently administered from April to June 2021, employing stratified sampling. We divided each city into different areas and then formed groups of 3–5 people to conduct surveys in locations such as parks, shopping centers, etc. The selection criteria of possible respondents are adults who have the ability to pay for their preferences. For instance, we approached the adults near rice displays in supermarkets to ask if they were willing to participate. After the survey was completed, questionnaires that lacked key information or exhibited evident inconsistencies were excluded from the analysis. This process yielded a total of 1714 valid responses, comprising 506 samples from Beijing (29.52%), 329 samples from Luoyang (19.19%), 406 samples from Wuhan (23.69%), and 470 samples from Shenzhen (27.42%). Statistical analysis demonstrated a proportional distribution of samples, rendering them representative of the urban population in these regions and facilitating subsequent in-depth empirical analysis.

### 3.2. Descriptive Statistics

The demographic and socioeconomic characteristics of the sample are presented in Table 2. The sample group from this study exhibits a gender ratio consistent with the desired sample characteristics: a higher level of education, a predominant representation of middle-aged individuals, married participants, and individuals from small-sized families with moderate income levels. These characteristics align with the consumer profile of the surveyed urban areas, thereby indicating that the sample is representative in terms of demographic and socioeconomic traits.

### 3.3. Variable Design and Measurement

#### 3.3.1. Dependent Variables

**Figure 1:**
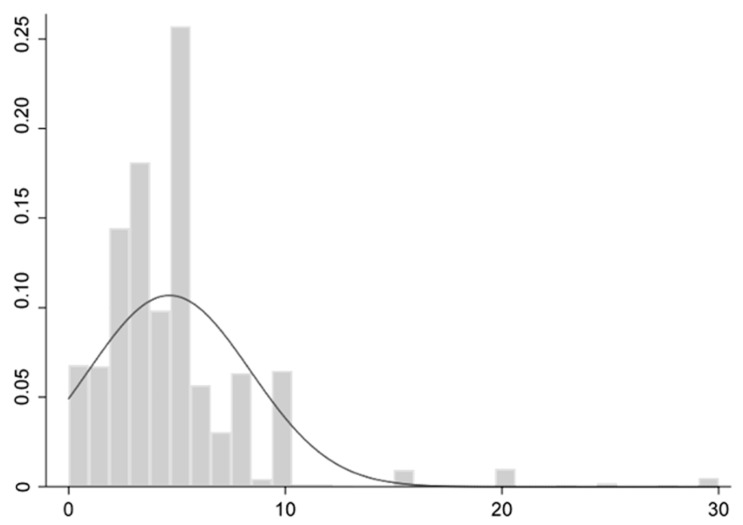
Distribution of premium payment prices.

In the study, design, Model 1 utilizes a five-point Likert scale to measure consumers’ willingness to pay a premium for higher-quality rice, which serves as the dependent variable. We asked the respondents “Will you pay a higher price for high-quality rice” and the Likert scale ranges from 1 (“Very unwilling”) to 5 (“Very willing”). Model 2 employs the actual amount consumers are willing to pay in addition to premium rice, referred to as the premium payment price. The distribution of the additional price consumers are willing to pay for premium rice is illustrated in Figure 1, which demonstrates a right-skewed distribution. The dependent variable Y is generated through the application of 5% Winsorization to the extreme values on the right tail.

#### 3.3.2. Independent Variables

For Model 1, the independent variables are structured around three core dimensions: product preference, cognitive perception, and purchasing experience. Each dimension comprises numerous secondary indicators, necessitating dimensionality reduction through Principal Component Analysis (PCA). Initially, the suitability of the data for factor analysis is assessed using the Kaiser-Meyer-Olkin (KMO) test and Bartlett’s test of sphericity [65,66]. The evaluation process mirrors the aforementioned validity analysis: a KMO value exceeding 0.9 indicates excellent suitability for factor analysis, values between 0.8 and 0.9 suggest very good suitability, values ranging from 0.6 to 0.8 indicate moderate suitability and values below 0.5 suggest inadequacy for factor analysis [67]. In this study, the KMO values for all three subscales exceed 0.6, and the *p*-values are significant, indicating that the data are suitable for factor analysis. Furthermore, factors with eigenvalues greater than 1 were extracted as common factors. As detailed in Table 3, each dimension—product preference factors, cognitive perception factors, and purchasing experience factors—yielded a single common factor. The independent variables within each dimension exhibited a variance explanation rate of approximately 60%, indicating a satisfactory level of explanatory power.

In the first stage of the study, the extracted common factors were utilized as core independent variables. Subsequently, the secondary indicators derived from the significant primary indicators identified in the first stage were employed as independent variables in the second stage. A hedonic pricing model was applied to empirically analyze the amounts that consumers are willing to pay for specific product attributes influenced by these factors, thereby elucidating the implicit prices associated with various attributes of rice products.

### 3.4. Methods

#### 3.4.1. Model for Estimating the Consumption Decision-Making Process

The Logit regression model, a generalized linear regression analysis framework, is particularly suited for analyzing non-continuous variables and is extensively utilized in the examination of influencing factors within social sciences, especially concerning individual decision-making behaviors [68,69]. Utilizing sample data collected from surveys, the relationship between independent and dependent variables is analyzed to ascertain which independent variables significantly affect variations in the dependent variable. The function form is as follows, in which $exp[u]=e^{u}$:

(1) $$\;P=\frac{1}{1+exp[-(a+bx)]}$$

The multivariate linear combination $a+b_{1}x_{1}+b_{2}x_{2}+b_{3}x_{3}+\cdots \ldots +b_{k}x_{k}$ could be represented by $\sum b_{i}x_{i}$, and simplifying the probability function:

(2) $$ln\frac{p}{1-p}=\sum b_{i}x_{i}$$

The general Logit function form is like below:

(3) $$logit\;(p)=ln\frac{p}{1-p}=\sum b_{i}x_{i}=a+b_{1}x_{1}+b_{2}x_{2}+b_{3}x_{3}+\cdots \ldots +b_{k}x_{k}$$

where *p* is the probability that *y* = 1, $B=b_{1},b_{2},{\ldots b}_{K}$ is the regression coefficient.

Depending on the research subject, binary, multinomial, and ordinal Logit models can be employed [70]. In the first stage, the variable indicating whether consumers are willing to pay a higher price for premium rice is an ordinal categorical variable, containing multiple ordered items. Therefore, an ordinal Logit regression model is used to analyze the factors influencing consumers’ willingness to pay a premium for high-quality rice. This model is more appropriate for handling multi-category dependent variables than binary models, fully utilizing the information available in the data [71]. The ordinal Logit model accounts for differences in the ranking of independent variables’ impacts on the dependent variable, providing results that differ from linear probability models and eliminating the influence of ranking on parameter estimation [72].

#### 3.4.2. Model for Estimating the Characteristic Price

In the second stage, based on Lancaster’s consumer theory [73], product attributes are used as independent variables to analyze their impact on consumers’ premium payment prices and to uncover the implicit market prices. A hedonic model is applied to examine how product characteristic attributes affect consumer utility, reflecting the market premium levels of different quality characteristics of premium rice.

The common functional forms of the hedonic price model include linear, semi-logarithmic, and double-logarithmic [74]:

(4) $$\mathrm{Linear}\;\mathrm{form}:\;P=\alpha +\sum P_{i}Z_{i}+\varepsilon$$

(5) $$\mathrm{Semi}\text{-}\mathrm{logarithmic}\;\mathrm{form}:\;lnP=\alpha +\sum P_{i}Z_{i}+\varepsilon$$

(6) $$\mathrm{Double}\text{-}\mathrm{logarithmic}\;\mathrm{form}:\;lnP=\alpha +\sum P_{i}{lnZ}_{i}+\varepsilon$$

In the model, *P* is the price of the product, α represents the sum of prices composed of other influencing factors except the considered dependent variable, $Z_{i\;}\;$ represents the *ith* feature variable, and ε represents the random error term. Since most dependent variables in this study are binary, the analysis primarily focuses on linear and semi-logarithmic forms.

## 4. Results

### 4.1. Factors Influencing the Consumer Decision-Making Process

#### 4.1.1. Baseline Regression Results of the First Stage

The first stage of the analysis investigates factors influencing the willingness to pay a premium. The frequency statistics of the dependent variable, presented in Table 4, indicate a higher concentration among respondents who are generally or relatively willing to pay. With an overall mean exceeding 3, a strong willingness to purchase premium rice is suggested, indicating the feasibility of promoting higher-quality, higher-priced rice.

The effectiveness of the ordered Logit regression model was initially assessed using a likelihood ratio test, leading to the rejection of the null hypothesis (*p* < 0.01). This finding establishes the validity of the independent variables incorporated into the model, thereby confirming the significance of the model’s construction.

As shown in Table 5, the regression results confirm *p* < 0.01, establishing the statistical significance of the model. The McFadden R-squared value of 0.165 indicates that the independent variables explain 16.5% of the variation in the willingness to pay a premium.

The regression coefficient for product characteristic preferences is 0.443 (*p* < 0.01), indicating that attributes such as appearance, origin, taste, and nutritional value of rice significantly enhance the willingness to pay a premium. This finding underscores the critical role of rice product characteristics in consumer decisions regarding premium pricing, thereby providing preliminary support for hypothesis H1.

The consumer perception and cognition factor have a regression coefficient of 0.286 (*p* < 0.01). This implies that consumer awareness—encompassing attention to nutritional labels, quality levels, and recognition of agricultural product labels—significantly and positively impacts their willingness to pay more. This outcome supports hypothesis H2, demonstrating that consumer beliefs intricately affect their propensity to pay higher prices for premium rice.

The purchasing experience factor exhibits a regression coefficient of 0.959 (*p* < 0.01), suggesting that factors such as consumer satisfaction with price-quality alignment, trust in rice quality and safety, and ease of assessing product quality during purchase exert substantial positive impacts on consumers’ willingness to pay a premium. This finding provides preliminary evidence supporting hypothesis H3, indicating that shopping satisfaction significantly influences payment willingness.

Pertaining to personal demographic characteristics, both education level and income markedly influence willingness to pay. The regression coefficient for education level is 0.422 (*p* < 0.01), indicating that higher educational attainment positively affects the willingness to pay a premium. Additionally, notable variations in premium rice consumption willingness exist among residents of different cities. The regression coefficient for household monthly income per capita is 0.232 (*p* < 0.01), suggesting that a higher household income correlates positively with the willingness to pay a premium.

In summary, consumer preferences regarding products, cognitive perception, and purchasing experience significantly influence their willingness to pay a premium. Among these factors, purchasing experience demonstrates the largest impact, particularly concerning the perceived association between price and quality. Enhancing consumer confidence in the alignment of quality and price, along with assurances regarding product safety, will substantially increase their willingness to pay higher prices for premium rice.

#### 4.1.2. Robustness Test of the First Stage

To test the robustness of the above results, alternative models were considered. Given the potential multicollinearity in the cross-sectional data, stepwise regression was used for comparison. The results, shown in Table 6, still indicate significant variables, such as product characteristic preferences, with directions consistent with the original model. Different regression methods do not alter the main conclusions of the study, and the hypotheses remain valid, suggesting that the estimated results are robust.

### 4.2. Characteristic Price Estimation

#### 4.2.1. Baseline Regression Results of the Second Stage

In the second stage, both linear and semi-logarithmic models were utilized to empirically analyze the prices consumers are willing to pay for product attributes influenced by the previously discussed factors, thereby exploring the underlying price dynamics associated with rice product characteristics. Following the removal of outliers, the dependent variable exhibited a minimum willingness to pay a premium of 0 yuan and a maximum of 15 yuan for 500 g of rice, with an average of 4.52 yuan and a standard deviation of 3.04. On average, consumers demonstrated a willingness to pay an additional 4.52 yuan per 500 g for rice of sufficiently high quality.

Regarding the distribution of independent variables, factors such as product characteristics, consumer cognitive perceptions, and purchasing experiences were found to significantly influence the willingness to pay a premium in the ordered Logit regression. Consequently, secondary indicators drawn from the pertinent primary indicators were employed as independent variables in the characteristic price models. Their distribution is detailed in Table 7.

Following the correlation analysis, it was observed that the relationships between premium prices and explanatory variables achieved statistical significance, with several core explanatory variables exhibiting correlation coefficients exceeding 0.7. To mitigate potential multicollinearity among the explanatory variables, ridge regression was employed for the empirical analysis of the sample data.

Various data processing methods can address multicollinearity, including stepwise regression, ridge regression, and Lasso regression [75,76]. Stepwise regression serves as a heuristic approach that circumvents the inclusion of collinear variables without directly resolving the underlying issues. Conversely, ridge regression and Lasso regression directly address collinearity by modifying the loss function; ridge regression utilizes L2 regularization while Lasso regression utilizes L1 regularization. Ridge regression is typically favored for resolving collinearity problems due to its enhanced robustness compared to ordinary least squares (OLS) regression. Consequently, this study selected ridge regression as the preferred method.

**Figure 2:**
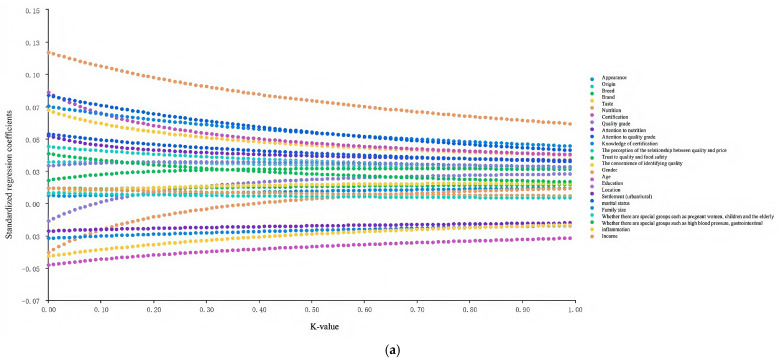

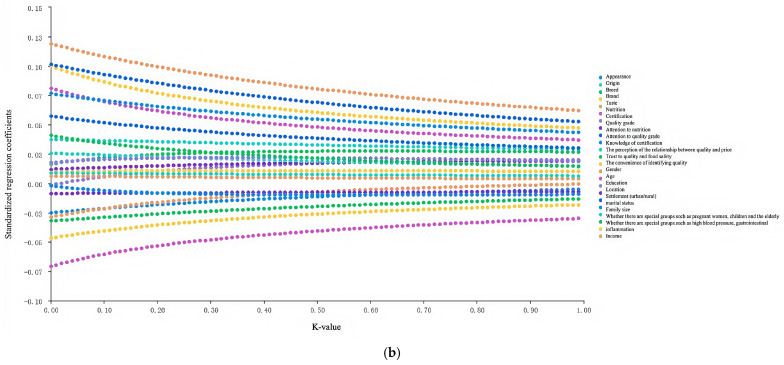
Ridge trace plots. (**a**) Linear model ridge trace diagram; (**b**) semi-logarithmic model ridge trace diagram.

The optimal K-value was determined by analyzing ridge trace plots (Figure 2), where the K-value of approximately 0.76 indicated a point at which the standardized regression coefficients of the independent variables stabilized. Therefore, the optimal K-value was established at 0.76, and the regression results for both the linear and semi-logarithmic models are presented in Table 8.

The econometric results indicate that both the linear and semi-logarithmic models passed the F-test, thereby confirming model validity. Additionally, a multicollinearity test revealed that the linear model exhibited a superior fit compared to the semi-logarithmic model. Specifically, the R^2^ and adjusted R^2^ values for the linear model are 0.111 and 0.098, respectively, while for the semi-logarithmic model, these values are 0.081 and 0.067. Consequently, the subsequent analysis concentrates on the results derived from the linear model. In the linear model, factors such as consumer emphasis on product characteristics—including origin, variety, brand, nutritional value, certification labels, and quality grade—along with consumer perceptions of nutritional labels, label recognition, and price-quality association, significantly and positively influence the premium price consumers are willing to pay. Notably, older consumers exhibit a lower willingness to pay premiums, whereas individuals with higher education levels, residents of southern cities, and those with greater household per capita income are more inclined to pay elevated premiums.

The regression coefficients associated with product characteristics—origin, variety, brand, nutritional value, certification labels, and quality grade—demonstrate significant impacts on the premium price. Specifically, for each level of improvement in these aspects, consumers are willing to pay an additional 0.066, 0.064, 0.098, 0.105, 0.052, and 0.095 yuan per 500 g, respectively. Among these factors, consumers display the highest willingness to pay a premium for nutritional value, followed closely by brand and quality grade.

Regarding consumer cognitive perception, each unit increase in consumer attention to nutritional labels correlates with an increase in their willingness to pay an additional 0.171 yuan per 500 g. Furthermore, for each additional certification label recognized on rice packaging, consumers are willing to pay an additional 0.106 yuan per 500 g. In terms of the shopping experience, each unit increase in consumer confidence regarding the quality-price alignment in the market leads to an increase in their willingness to pay an additional 0.086 yuan per 500 g.

#### 4.2.2. Robustness Test of the Second Stage

To account for potential heteroscedasticity in the cross-sectional data, quantile regression (QR) was employed for robustness testing. QR, which is conceptually similar to linear ordinary least squares (OLS) regression, does not necessitate the assumption of normality for the dependent variable and is robust to outliers and heteroscedasticity [77], making it suitable for the robustness analysis of regression models. Three models were estimated at quantile points of 0.25, 0.50, and 0.75, with results presented in the corresponding table.

As shown in Table 9, product attributes—including nutritional value, consumer attention to nutritional labels, label recognition, and consumer perception of price-quality association—significantly affect premium prices at different quantiles. Notably, as premium prices increase, attention to nutritional value rises significantly. Consumers at the median quantile are more influenced by nutritional label information and price-quality associations compared to those at other quantiles. Moreover, an increase in consumer recognition of labels has the greatest impact on those at the 0.75 quantile. The R^2^ of the original linear model is higher than that of the other quantiles, indicating a superior overall fit.

## 5. Discussion

### 5.1. Key Findings

This study constructed an indicator system to evaluate factors influencing consumers’ willingness to pay a premium, encompassing three dimensions: product preferences, cognitive perceptions, and purchasing experiences. Field research conducted across four representative cities resulted in 1714 valid questionnaires, providing the microdata necessary for empirical analysis. The findings of this study elucidate critical determinants underlying consumers’ willingness to pay (WTP) a premium for rice products, thereby offering significant implications for agricultural marketing and behavioral economics research. Through the integration of hedonic pricing theory, the Howard-Sheth model, and the Theory of Planned Behavior, the proposed hypotheses were validated, and novel mechanisms in premium pricing dynamics were demonstrated. Below, the results are interpreted within the theoretical framework, juxtaposed with existing literature, and their practical and methodological implications are discussed.

#### 5.1.1. Product Characteristics as Primary Drivers

The observed dominance of product attributes on WTP, specifically nutritional value (β = 0.105), brand (β = 0.098), and quality grade (β = 0.095), aligns closely with hedonic pricing theory, which posits that consumers implicitly assign prices to individual product characteristics [29]. Consequently, these findings substantiate H1 by demonstrating the significant role of both intrinsic-like breeds (β = 0.064), and extrinsic-like origin (β = 0.066) attributes in augmenting WTP. Notably, the premium associated with nutritional value underscores a global trend towards health-oriented consumption patterns, echoing prior observations in organic food markets [78]. By explicitly linking attribute-specific premiums, such as ¥0.105/500 g for nutritional value, this research provides granular insights into consumer valuation processes.

#### 5.1.2. Cognitive Perception and Purchasing Experience

The significant influence of nutritional label awareness (β = 0.171) and certification knowledge (β = 0.106) corresponds with cognitive evaluation mechanisms proposed in the Theory of Planned Behavior [45], thereby validating H2. Highlights the importance of improving nutritional awareness and knowledge of labels. Moreover, the substantial impact of purchasing experience extends the Howard-Sheth model [50], elucidating how post-purchase satisfaction dynamically reshapes consumers’ future purchasing intentions, thus confirming H3. According to the existing literature review [79], standardized certification labels enhance consumer trust and mitigate perceived risks, facilitating the willingness to pay premium prices. Therefore, experiential feedback reasonably emerges as a pivotal factor in shaping sustained consumer valuation behaviors.

#### 5.1.3. Demographic Heterogeneity

The positive correlation of education (β = 0.115) and income (β = 0.160) with WTP is consistent with socioeconomic theories related to luxury consumption, which posit that affluent and well-educated consumers typically prioritize quality over price considerations [80]. Conversely, older consumers exhibit resistance towards premium payments, possibly due to generational differences in price sensitivity or established preferences for traditional rice varieties. This finding aligns with cultural studies emphasizing age-related consumer preferences in staple foods [81].

### 5.2. Primary Contribution

This study’s innovations arise from the synergistic integration of three distinct theoretical frameworks, thereby addressing critical limitations observed in prior research. First, leveraging hedonic pricing theory enabled the explicit decomposition of product value into attribute-specific premiums, providing enhanced empirical granularity that prior research often neglected. Second, this study adopts a two-stage analytical framework. In the first stage, structural hypotheses (H1–H3) are validated. In the second stage, attribute-level premiums are quantified. For instance, promoting one unit of attention to nutrition facts causes the premium by ¥0.171/500 g, and knowledge of one more certification leads to an increased WTP by ¥0.106/500 g. This approach addresses the deficiency noted by Lancaster in the hedonic pricing literature, where decision-making and premium quantification were frequently conflated [73]. This methodological clarity significantly advances the understanding of premium valuation processes. Furthermore, the application of the Howard-Sheth model was expanded by incorporating post-purchase evaluations into consumer behavior analysis, emphasizing the role of experiential feedback in shaping consumer intentions, a perspective previously underexplored.

Notably, while nutritional value is initially highly regarded by consumers, limited attention to nutritional labeling indicates a disconnect between cognitive recognition and actionable purchasing decisions. This observation notably challenges the assertion that cognitive determinants dominate WTP in food markets [82]. Therefore, policy interventions aimed at enhancing consumers’ nutritional label literacy or simplifying labeling systems are crucial for bridging this cognitive-action gap. Such dual-focused insights hold substantial practical implications for policymakers and industry stakeholders alike.

## 6. Conclusions

### 6.1. Conclusions and Implication

This study elucidates the underlying mechanisms of quality-price equilibrium formation within rice markets in developing economies, demonstrating that consumer willingness to pay (WTP) a premium is systematically influenced by hierarchical factors extending from intrinsic product attributes to post-purchase experiential feedback loops. By integrating consumer decision-making theories with a robust empirical framework, the findings advance the understanding of premium pricing mechanisms, thus enabling the proposal of actionable strategies that align production standards with consumer valuations. Notably, the quantification of implicit attribute-specific prices—such as attention to nutrition fact (¥0.171/500 g), knowledge of certification (¥0.106/500 g), and nutrition value (¥0.105/500 g)—provides a rigorous, data-driven foundation facilitating market coordination optimization for stakeholders. Consequently, specific implications emerge for policymakers, supply chain managers, and marketers, as delineated below.

#### 6.1.1. Policy Implications

Evidence from our study indicates that directly linking nutrition value to market pricing could bring a higher premium on rice. Policymakers should prioritize the revision of national quality metrics by integrating consumer-valued attributes (like nutrition) into existing standards. Furthermore, it is found that consumers are willing to pay for high nutritional value but do not have the ability to identify relevant information, which hinders the conversion from nutritional value to premium payment. Thus, simplifying certification systems through standardized, visually intuitive labels (e.g., “Nutrition Star Rating”) will mitigate information asymmetry. Concurrently, mandating retailer education programs, and government-funded campaigns targeting vulnerable populations can further enhance nutritional literacy and label comprehension.

#### 6.1.2. Supply Chain Implications: Prioritizing Attributes with High Premium Potential

Supply chain managers should strategically invest in consumer-oriented breeding programs that prioritize high-premium traits, especially the breed that balances nutrition and tastes well. To achieve this, collaborations with agronomists are recommended to develop rice varieties that effectively balance yield and quality. In addition, as consumers have a significantly higher willingness to pay a premium for labels, strengthening certification partnerships with authoritative entities (e.g., Organic Food Certification Center) to validate high-value claims can elevate consumer WTP. The perception of a high correlation between quality and price is so vital to the purchasing experience, suggesting the significance of embracing advanced technological solutions. The development of blockchain platforms for transparent supply chain tracking, as exemplified by E-commerce platform’s rice traceability system, can further optimize quality-price signaling through dynamic pricing models.

#### 6.1.3. Marketing Implications

Due to the significant effect of purchasing experience, marketers should leverage experiential feedback mechanisms. Specifically, they can emphasize explicit quality-price alignment guarantees using interactive strategies, like in-store tastings and augmented reality (AR)-enabled packaging. Moreover, employing QR codes to illustrate farming practices and display the livestream of farming could consequently increase premium rice sales. Given the significant role of trust identified in our findings, highlighting consistent post-purchase benefits is suggested; for instance, stable taste profiles across product batches would demonstrably reinforce consumer trust. Strategically segmenting markets is equally essential; it is recommended to target health-conscious urban consumers with nutrition-centered campaigns, and to tailor regional strategies based on the geographic preference. Amplifying digital engagement through targeted social media initiatives is also an effective way to enhance the consumer purchasing experience.

Collectively, this study not only demonstrates the hierarchical influence of product attributes, cognitive perceptions, and consumer experiences on premium pricing dynamics but also effectively translates these theoretical insights into targeted, actionable strategies for diverse stakeholders. By aligning regulatory standards with consumer priorities, optimizing attribute-centric supply chain practices, and effectively crafting consumer experiential narratives, stakeholders can systematically bridge existing quality-price gaps, thus fostering sustainable agricultural value chains. This study offers replicable and adaptable premium pricing strategies for other staple crops within emerging economies.

### 6.2. Limitations and Future Directions

While this study provides valuable insights into the determinants of consumers’ willingness to pay (WTP) a premium for rice products, several limitations must be acknowledged to contextualize the findings and guide future research endeavors.

#### 6.2.1. Geographical Representativeness

The findings indicate substantial heterogeneity among different regions. Although the four research sites were selected as rigorously and scientifically as possible in accordance with different directions and different economic levels, different sizes, dietary habits, etc., the conclusions of the study may be biased, which is a limitation of this study. Therefore, expanding the research scope, for instance, by including sampling surveys in the northeastern region, may yield new insights. Comparing findings across various regions could provide valuable references for product development strategies. Future studies should conduct data collection and surveys in a wider range of regions to enhance the generalizability of the conclusions.

#### 6.2.2. Temporal and Contextual Limitations

Several studies have shown that COVID-19 pandemics may have a sustained impact on market shocks and may also have an impact on consumer behavior [83,84]. The pandemic may affect consumers’ food purchasing habits, willingness to pay, and especially changes in consumer confidence, price sensitivity, and purchasing behavior. This is despite the fact that normal communication activities had resumed in the offline study city at the time of data collection for this study. And the results of this study are largely consistent with the results of studies conducted during non-special periods, probably because rice is a necessity for consumers and even a larger premium, which would not have a significant impact on Engel’s coefficient. But the pandemic may still have had an impact on consumer behavior, and factors such as supply fluctuations in vertical retail channels deserve attention in subsequent longitudinal and comparative studies. In the future, if possible, some follow-up studies could be conducted to better capture the possible impact of a major public crises on the implementation of a high-priced, high-value market.

#### 6.2.3. Innovative Research Methods

Traditional stated preference surveys have been proven to have a shortcoming regarding hypothesis bias [85]. Future research could benefit from triangulating findings with experimental auctions under controlled market conditions to validate consumer WTP estimates, and implementing behavioral choice experiments using actual products. Along with gathering information on consumers’ revealed preferences, such as scanned data, could enable valuable comparative analyses.

## Figures and Tables

**Table 1:** Characteristics of surveyed cities.

City	Region	Population Size (million)	GDP (¥/capita)	Engel’s Coefficient for Urban Residents (%)	Grain Food Consumption of Urban Residents (kg/capita)
Beijing	North	2189.0	183,980.0	21.0	106.0
Luoyang	Central	752.5	76,219.0	26.4	133.9
Wuhan	Central-south	1364.9	135,251.0	29.9	116.6
Shenzhen	South	627.9	174,542.0	31.7	94.5
China	Total	141,260.0	83,111.0	28.6	124.8

Data source: China Statistical Yearbook 2022 [59], China Rural Statistical Yearbook 2022 [60], Beijing Statistical Yearbook 2022 [61], Henan Statistical Yearbook 2022 [62], Hubei Statistical Yearbook 2022 [63], Guangdong Statistical Yearbook 2022 [64]. Provincial-level data were utilized as proxies where finer-grained statistics were unavailable.

**Table 2:** Demographic characteristics of the survey sample (Unit: person, %).

Project	Class	Samples	Percentage
Gender	Male	637	37.16%
	Female	1077	62.84%
Age	Under 18	15	0.88%
	18~29	624	36.41%
	30~45	690	40.26%
	46~65	308	17.97%
	Above 65	77	4.49%
Education	Junior high school or below	120	7.00%
	High school (including secondary school)	301	17.56%
	Undergraduate (including college)	1074	62.66%
	Postgraduate or above	219	12.78%
Location	Beijing	506	29.52%
	Luoyang	329	19.19%
	Wuhan	406	23.69%
	Shenzhen	473	27.60%
Marriage	Single	606	35.36%
	Married (including divorced or widowed)	1108	64.64%
Household size	≤1 person	61	3.57%
	2~4 people	1216	71.07%
	More than four people	434	25.37%
Whether there are pregnant women/children/elders at home	Yes	958	55.89%
	No	756	44.11%
Whether there are chronic patients at home	Yes	636	37.11%
	No	1078	62.89%
Monthly personal income	Less than 2000 yuan	68	3.97%
	2000~5000 yuan	439	25.61%
	5000~10,000 yuan	572	33.37%
	10,000~15,000 yuan	319	18.61%
	15,000~20,000 yuan	175	10.21%
	More than 20,000 yuan	141	8.23%
Sample size	1714		

Data source: data compiled from questionnaire survey.

**Table 3:** Principal component analysis results.

Variable Classification	Variable Name	Coefficient of Load	Charact-Eristic Root	Variance Cumulative Explanation Rate (%)
Product preferences	Appearance	0.669	4.729	59.113
	Origin	0.745		
	Breed	0.811		
	Brand	0.786		
	Taste	0.734		
	Nutrition	0.771		
	Certification	0.817		
	Quality grade	0.805		
Cognitive perception	Attention to nutrition fact	0.869	1.964	65.465
	Attention to quality grade	0.876		
	Knowledge of certification	0.664		
Purchasing experience	The perception of the relationship between quality and price	0.744	1.840	61.320
	Trust to quality and food safety	0.831		
	The convenience of identifying quality	0.772		

**Table 4:** Ordered Logit regression analysis of dependent variable frequency distribution.

Variable Name	Option	Frequency	Percentage	Mode	Median	Mean	Standard Error
Willingness to pay premium	Very Unwilling = 1	86	5.02%	4	4	3.51	1.03
	Unwilling = 2	138	8.05%				
	Neutral = 3	436	25.44%				
	Willing = 4	879	51.28%				
	Very Willing = 5	175	10.21%				
	Total	1714	100.00%				

**Table 5:** Ordered Logit regression analysis results.

Variable Name	Regression Coefficient (*z*-Value)	Standard Error	Waldχ^2^	*p*-Value	OR Price	OR Value 95%CI
Product preference	0.443 *** (7.059)	0.063	49.829	0.000	1.557	1.377~1.760
Cognitive perception factors	0.286 *** (5.119)	0.056	26.201	0.000	1.331	1.193~1.484
Purchasing experience factors	0.959 *** (15.226)	0.063	231.827	0.000	2.608	2.305~2.950
Gender	−0.057 (−0.574)	0.100	0.329	0.566	0.944	0.777~1.148
Age	−0.101 (−1.318)	0.077	1.736	0.188	0.904	0.778~1.050
Education	0.422 *** (5.312)	0.080	28.220	0.000	1.526	1.305~1.783
Location	−0.149 *** (−3.365)	0.044	11.320	0.001	0.862	0.790~0.940
Marriage	−0.199 (−1.418)	0.140	2.012	0.156	0.819	0.622~1.079
Household size	0.006 (0.192)	0.031	0.037	0.848	1.006	0.946~1.070
Whether there are pregnant women/children/elders at home	−0.135 (−1.230)	0.110	1.514	0.219	0.873	0.704~1.084
Whether there are chronic patients at home	−0.096 (−0.933)	0.103	0.870	0.351	0.908	0.742~1.112
Monthly personal income	0.232 *** (5.636)	0.041	31.767	0.000	1.261	1.164~1.368
McFadden R^2^: 0.165						

Note: *** indicates that the test is passed at the significance level of 1%.

**Table 6:** Robustness test results.

Variable	Original Model	Stepwise Regression Model
Product preference	0.443 *** (7.059)	0.199 *** (8.366)
Cognitive perception factors	0.286 *** (5.119)	0.107 *** (4.820)
Purchasing factors	0.959 *** (15.226)	0.391 *** (16.937)
Age	−0.101 (−1.318)	−0.074 *** (−3.083)
Education	0.422 *** (5.312)	0.181 *** (5.858)
Location	−0.149 *** (−3.365)	−0.059 *** (−3.414)
Monthly personal income	0.232 *** (5.636)	0.070 *** (4.395)
Adjusted R−squared	0.165	0.401

Note: *** indicates that the test is passed at the significance level of 1%.

**Table 7:** Indicator system and variable description.

Primary Indicators	Variable Name	Variable Assignment	Mode	Median	Mean	Standard Deviation	Expected Direction
Product preferences	Appearance	Very unimportant = 1 Unimportant = 2 Neutral = 3 Important = 4 Very important = 5	4	4	3.398	1.090	+
	Taste		5	5	4.435	0.913	+
	Nutrition		5	5	4.264	1.006	+
	Origin		3	3	3.272	1.184	+
	Breed		4	4	3.465	1.137	+
	Brand		3	3	3.373	1.115	+
	Certification		5	4	3.975	1.061	+
	Quality grade		5	5	4.258	0.955	+
Cognitive perception	Attention to nutrition fact	Never = 1 Rarely = 2 Occasionally = 3 Often = 4 Always = 5	3	3	2.694	1.122	+
	Attention to quality grade		3	3	2.954	1.171	+
	Knowledge of certification	The actual number of identifiers recognized	2	2	2.047	1.219	+
Purchasing experience	The perception of the relationship between quality and price	Completely disagree = 1 Disagree = 2 Neutral = 3 Agree = 4 Completely agree = 5	4	2	3.459	1.008	+
	Trust to quality and food safety	Strongly distrust = 1 Distrust = 2 Neutral = 3 Trust = 4 Strongly trust = 5	4	4	3.547	0.961	+
	The convenience of identifying quality	Very inconvenient = 1. Inconvenient = 2. Neutral = 3; Convenient = 4; Very convenient = 5	3	3	2.917	1.008	+

**Table 8:** Ridge regression estimation results.

Variable Name	Regression Coefficient	
Dependent variable	Y	Ln_Y
Constant	0.651 (1.299)	0.685 *** (5.303)
Appearance	0.026 (0.774)	−0.005 (−0.628)
Origin	0.066 ** (2.225)	0.016 ** (2.295)
Breed	0.064 ** (2.164)	0.015 ** (2.070)
Brand	0.098 *** (3.191)	0.029 *** (3.943)
Taste	0.028 (0.779)	−0.001 (−0.102)
Nutrition	0.105 *** (3.144)	0.027 *** (2.911)
Certification	0.052 * (1.652)	0.011 (1.372)
Quality grade	0.095 *** (2.780)	0.013 (1.316)
Attention to nutrition fact	0.171 *** (5.191)	0.037 *** (4.854)
Attention to quality grade	0.040 (1.290)	0.008 (1.042)
Knowledge of certification	0.106 *** (3.313)	0.022 *** (3.145)
The perception of the relationship between quality and price	0.086 ** (2.228)	0.012 (1.195)
Trust to quality and food safety	0.040 (1.003)	−0.012 (−1.186)
The convenience of identifying quality	0.034 (0.878)	0.005 (0.517)
Gender	0.038 (0.460)	0.005 (0.264)
Age	−0.104 ** (−2.465)	−0.025 *** (−2.724)
Education	0.115 ** (2.258)	0.016 (1.472)
Location	0.121 *** (3.683)	0.031 *** (4.424)
Marriage	−0.125 (−1.634)	−0.014 (−0.837)
Household size	0.007 (0.283)	0.002 (0.432)
Whether there are pregnant women/children/elders at home	0.119 (1.538)	0.022 (1.344)
Whether there are chronic patients at home	−0.128 (−1.581)	−0.029 * (−1.665)
Monthly personal income	0.160 *** (5.322)	0.033 *** (5.153)
Sample size	1714	1619
R^2^	0.111	0.081
Adjust R^2^	0.098	0.067
F-value	F (24,1686) = 8.780, *p* = 0.000	F (24,1594) = 5.862, *p* = 0.000

Note: *, ** and *** indicate that the test is passed at the significance level of 10%, 5% and 1%, respectively.

**Table 9:** Quantile regression robustness test.

Variable Name	Percentile 0.25	Percentile 0.5	Percentile 0.75	Original Model
Constant	0.005 (0.006)	−0.456 (−0.540)	−0.231 (−0.226)	0.651 (1.299)
Appearance	−0.039 (−0.572)	−0.009 (−0.132)	−0.022 (−0.260)	0.026 (0.774)
Origin	0.149 ** (1.981)	0.089 (1.153)	0.176 * (1.832)	0.066 ** (2.225)
Breed	−0.058 (−0.694)	0.067 (0.756)	0.026 (0.226)	0.064 ** (2.164)
Brand	0.105 (1.255)	0.114 (1.343)	0.290 *** (2.773)	0.098 *** (3.191)
Taste	−0.119 (−1.172)	−0.063 (−0.625)	0.032 (0.267)	0.028 (0.779)
Nutrition	0.182 * (1.952)	0.202 ** (2.178)	0.220 * (1.950)	0.105 *** (3.144)
Certification	−0.042 (−0.449)	0.000 (0.005)	0.041 (0.355)	0.052 * (1.652)
Quality grade	0.135 (1.338)	0.184 * (1.767)	−0.011 (−0.086)	0.095 *** (2.780)
Attention to nutrition fact	0.291 *** (3.758)	0.350 *** (4.370)	0.289 *** (3.037)	0.171 *** (5.191)
Attention to quality grade	−0.081 (−1.032)	−0.199 ** (−2.545)	0.016 (0.167)	0.040 (1.290)
Knowledge of certification	0.126 ** (2.275)	0.128 ** (2.199)	0.168 ** (2.348)	0.106 *** (3.313)
The perception of the relationship between quality and price	0.203 *** (2.762)	0.230 *** (3.099)	0.181 * (1.954)	0.086 ** (2.228)
Trust to quality and food safety	−0.006 (−0.069)	−0.033 (−0.391)	0.021 (0.206)	0.040 (1.003)
The convenience of identifying quality	0.012 (0.169)	0.054 (0.719)	0.084 (0.915)	0.034 (0.878)
Gender	−0.006 (−0.044)	−0.067 (−0.508)	−0.082 (−0.502)	0.038 (0.460)
Age	−0.198 ** (−2.004)	−0.105 (−1.031)	−0.086 (−0.657)	−0.104 ** (−2.465)
Education	0.020 (0.191)	0.091 (0.853)	−0.029 (−0.228)	0.115 ** (2.258)
Location	0.063 (1.111)	0.144 ** (2.465)	0.290 *** (4.032)	−0.177 (−1.193)
Marriage	0.220 (1.232)	0.037 (0.201)	−0.313 (−1.333)	−0.125 (−1.634)
Household size	0.021 (0.418)	0.032 (0.789)	−0.003 (−0.069)	0.007 (0.283)
Whether there are pregnant/children/elders at home	0.038 (0.268)	0.223 (1.530)	0.494 *** (2.755)	0.119 (1.538)
Whether there are chronic patients at home	−0.110 (−0.854)	−0.229 * (−1.678)	−0.275 (−1.617)	−0.128 (−1.581)
Monthly personal income	0.072 (1.350)	0.158 *** (2.938)	0.281 *** (4.348)	0.160 *** (5.322)
Sample size	1714	1714	1714	1714
R^2^	0.094	0.083	0.063	0.111

Note: *, ** and *** indicate that the test is passed at the significance level of 10%, 5% and 1%, respectively.

## Data Availability

The data presented in this study are available on request from the corresponding author. The data are not publicly available due to privacy restrictions.

## Abbreviations

The following abbreviations are used in this manuscript:

WTP	Willingness to Pay
PCA	Principal Component Analysis
